# Complications of penile self-injections: investigation of 680 patients with complications following penile self-injections with mineral oil

**DOI:** 10.1007/s00345-017-2110-9

**Published:** 2017-10-28

**Authors:** Johannes Nordsteien Svensøy, Valentine Travers, Palle Jörn Sloth Osther

**Affiliations:** 1 Lege Svensøy, Oslo, Norway; 2 Centre de santé, Seine-Saint-Denis, Saint-Denis, France; 3 Urological Research Center (URC), Department of Urology, Lillebaelt Hospital, Fredericia, Denmark

**Keywords:** Penile self-injection, Self-administration, Penile injection, Penile augmentation, Paraffinoma, Granuloma, Lipogranuloma, Mineral oil, Penile induration, Penile swelling, Myanmar, Mae Tao Clinic

## Abstract

**Objective:**

Penile implants and injection of foreign materials have been described in texts like Kama Sutra for more than 1500 years, and are still being practiced around the world. The extent of this practice is unknown, and the documentation available today only scratches the surface. This study investigates and documents the complications after penile self-injections at the Mae Tao Clinic. To our knowledge, this study represents the largest series of patients representing complications to penile self-injections.

**Study design:**

Retrospective study.

**Methods:**

We investigated data on 680 patients admitted with penile self-injections during a 5-year period. Data were studied for general patient data, symptoms, time of injection, and treatment.

**Results:**

Age at admittance ranged from 17 to 68 with a mean age of 32 years. Time between injection and presentation was registered with a mean of 36.7 months, over half presented with complications within 1 year. Most frequent complications were penile pain (84%), swelling (82.5%), induration (42.9%), purulent secretion (21.8%), and ulceration (12.8%). Of the 680 patients, 507 (74.6%) underwent surgical treatment (503 excision and 4 circumcision), while 173 (25.4%) were treated conservatively.

**Conclusion:**

Our data suggest that penile self-injections with mineral oil are more prevalent in certain areas than previously acknowledged. In 5 years, more than 680 patients presented with complications to penile self-injections, of which 75% needed surgical intervention, mainly in the form of radical excision of the lesions followed by skin grafting. Preventive measures to this physically and psychologically devastating problem are highly warranted.

## Background

Penile implants and injection of foreign materials have been described in texts like Kama Sutra for more than 1500 years. Implants of glass, stone, bullets, ivory, gems, gold, plastic and other solid objects, as well as injections with silicone, paraffin, Vaseline, petroleum jelly [1], cod liver oil [2], nandrolone decanoate [3], waxes, and mineral oils for penile augmentation have been described in the literature.

Robert Gersuny was the first to describe injections of mineral oil as a medical procedure in 1899, injecting Vaseline to substitute the loss of testicles after tuberculosis epididymitis [4]. Since then mineral oil injections have been used for a wide range of cosmetic purposes, i.e., cleft palate, wrinkles, face deformities, baldness, and muscle, breast and penile augmentation [5, 6]. Heidingsfeld presented the first report of adverse effects of human body oil injections in 1906, describing disfiguring subcutaneous nodules after paraffin injections for facial wrinkles [4, 7]. In 1917 Fermiet and Fermiet reported similar tumours occurring weeks to years after injection of mineral oil [8]. After several reports of serious adverse effects, these treatment modalities were omitted in traditional medicine. Nevertheless, they are still used by non-medical personnel or as self-injections mostly for cosmetic purposes.

Penile self-injections are performed with the purpose of increasing the size of the penis. Most often mineral oils or mineral oil-like substances are used for this purpose. Complications occur due to the fact that human tissue lacks the enzymes to metabolize interstitial exogenous oils [9, 10]. This, results in the formation of a paraffinoma, also referred to as oleoma or sclerosing lipogranuloma, presenting as a characteristic histological lesion. Although there are no exact statistics available, literature suggest the procedure to be more commonly performed in Asia and Eastern Europe. An earlier study of 639 Burmese fishermen in Thailand revealed a prevalence of 7.5% [11]. Another study among Hungarian prisoners found that 15.7% had performed penile self-injections [12]. The aim was to investigate and document complications after penile self-injections to increase the knowledge and raise awareness about complications to established health-care takers, risk groups, and to the general population in a high prevalence area.

## Materials and methods

The study was performed at the Mae Tao Clinic, which is located on the Thailand–Myanmar border. It annually treats more than 100,000 patients from both sides of the border with free health-care [13]. The clinic was founded by Dr. Cynthia Maung in 1989. Data were collected from patients at the Surgery and Trauma department at the Mae Tao Clinic.

Data on penile self-injections were sampled retrospectively to explore prevalence, risks, and treatment modalities for management of complications. All data were analysed anonymously.

**Fig. 1:**
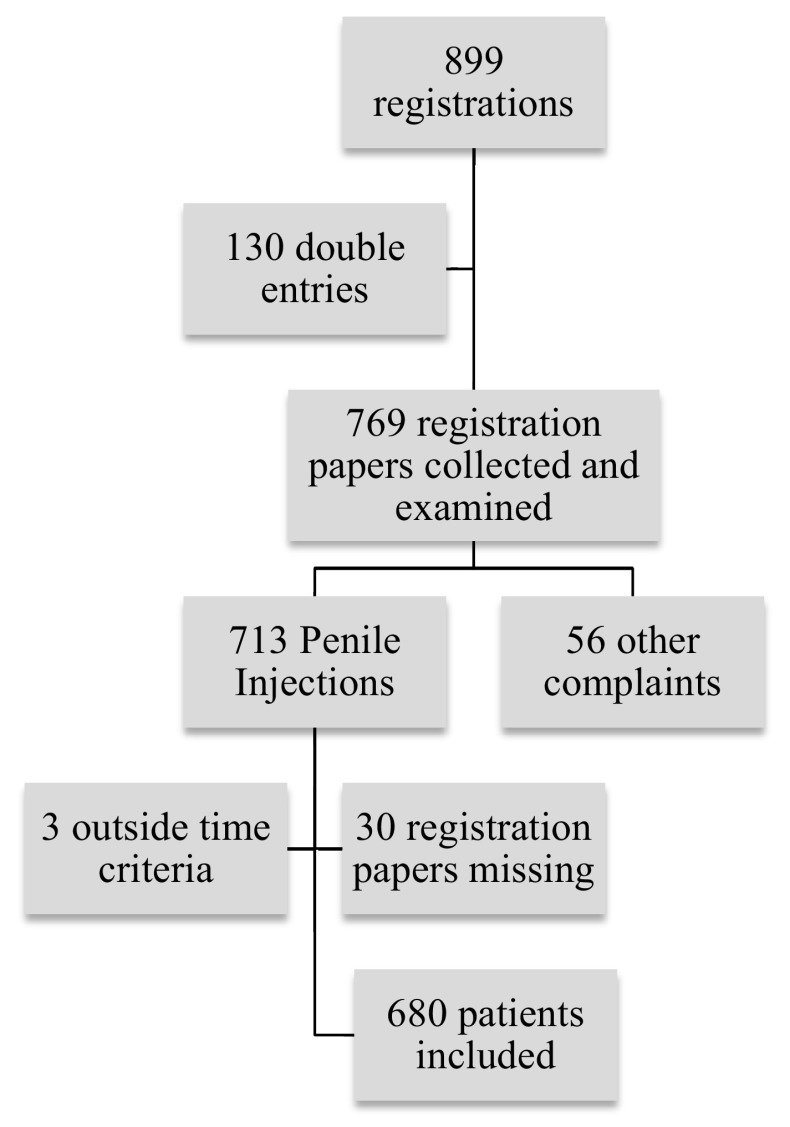
Included patients from the registration at the Mae Tao Clinic

All registered cases of penile injections at the Mae Tao Clinic from 2010 through 2014 were initially included, comprising a total of 899 registrations (Fig. 1). The list of patients was manually checked for errors and double entries (patients registered more than one time); 130 double entries were found and removed. The main reason for double entries was re-registration, when patients returned for skin grafts after excision, or when conservative treatment regimens were converted to surgery due to lack of improvement.

769 registration papers were collected from the archive and further investigated: 56 were wrongly registered as penile injections, and 30 registration papers were missing; 3 patients were excluded as the dates of complication presentation were outside the study period (2010–2014).

Finally, 680 patients were included and their registration papers studied for 20 predefined variables of complaints (Table 1). Additionally, age, weight, address, ethnicity, time of injection and treatment of complication were also registered. The 680 patients were grouped according to main complication into Mild, Moderate, Severe, and Life Threatening (Table 1). All data were anonymously registered in SPSS v.22.

**Table 1:** Predefined variables of complaints

Mild	Moderate	Severe	Life threatening
Penile pain	Phimosis	Induration	Fournier’s gangrene
Swelling	Ulceration	Necrosis	Sepsis
Penile erythema	Purulent secretion		
Itching at injection area	Pale penile skin colour change		
Discharge	Dysuria		
	Fever		
	Atrophy		
	Recurrent bleeding		

## Results

**Fig. 2:**
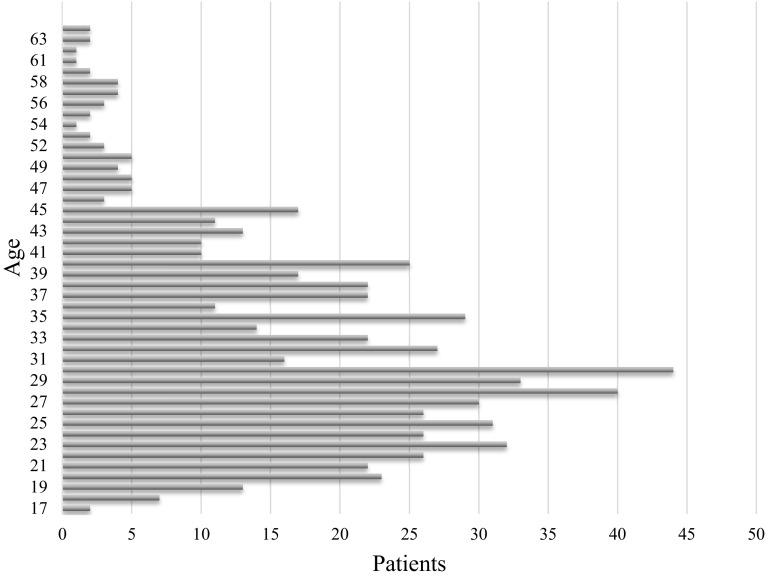
Age distribution of patients presenting with complications following penile self-injections

The mean age among the 680 patients was 32 years, ranging from 17 to 68 years (for distribution see Fig. 2). There was no significant difference in symptoms presented or treatment given in regard to age.

Time between injection and presentation of symptoms was registered in 643 patients with a mean of 36.7 months, ranging from a few days to 30 years: 346 (53.8%) presented to the Mae Tao Clinic with complications within 1 year after injection, 72 (11.2%) between 1 and 2 years, 47 (7.3%) between 2 and 3 years, 31 (4.8%) between 3 and 4 years, and 147 (22.9%) presented with complications more than 4 years after injection. The age of the patient at the time of injection ranged between 13 and 66 years (mean 29 years).

**Fig. 3:**
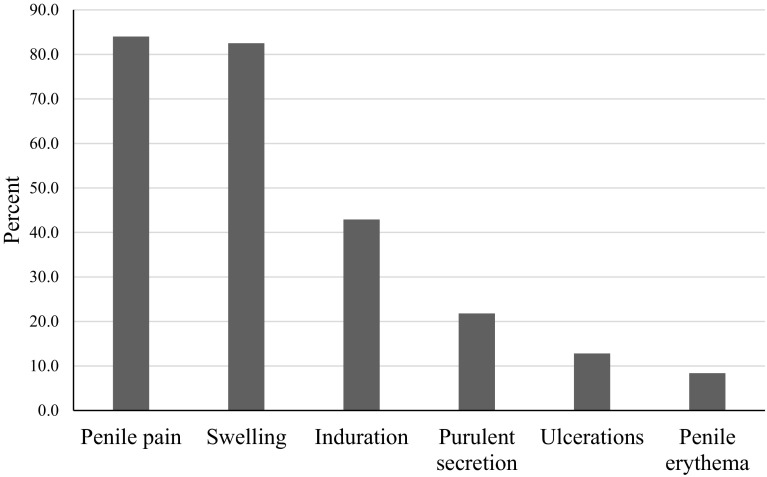
Presenting symptoms of penile self-injections

The most frequently reported symptoms were penile pain, swelling, induration, purulent secretion, and ulceration (Fig. 3, Table 2). Of these, pain was the most prominent, covering intermittent pain, pain during erection, and chronic pain.

**Table 2:** Presenting symptoms of penile self-injections

Symptom	Patients (*N* = 680)	Percent
Penile pain	571	84
Swelling	561	82.5
Induration	292	42.9
Purulent secretion	148	21.8
Ulcerations	87	12.8
Penile erythema	57	8.4

Moderate complications consisted of phimosis, ulcers, and purulent secretion (Table 1). Ulcers presented as chronic wounds. Of 87 patients with ulcerations, 42 had purulent secretion. Patients with ulcers had a mean presentation time of 48 months after injection compared to a mean of 36.7 months for the whole group. Atrophy of the skin was seen in 9 (1.3%) patients. Voiding complaints was seen in 28 (4.1%), including dysuria and stranguria; 35 (5.1%) of the patients had pale penile skin colour changes. Recurrent bleeding was reported in 3 patients.

Severe induration, which was seen in 292 (42.9%) patients, was grouped as a severe complication, since this finding most often needs surgical correction. Induration was noted as hard firm masses, which in the literature has been attributed to lipogranulomas. Necrosis was seen in 11 (1.6%) patients, 2 of which was admitted short time after injecting mineral oil (1 and 3 weeks).

Mode of treatments were grouped into conservative 173 (25.4%), and surgical 507 (74.6%) (Table 3). Conservative treatment consisted of dressing, and/or antibiotics, and/or painkillers. It is important to point out that the conservative treatment group also included patients reluctant to surgical treatment. Surgical treatment included complete excision of involved tissue. In 4 cases circumcision was sufficient to remove the affected lesion. All complete excisions were followed by a split thickness skin graft from the anterior thigh after 3 to 10 days. Surgical excision was done under penis block. General anaesthesia was not available at the Mae Tao Clinic.

**Table 3:** Treatment performed at the Mae Tao Clinic

	Frequency	Percent
Surgical treatment	507	74.6
Conservative treatment	173	25.4
Total	680	100

**Fig. 4:**
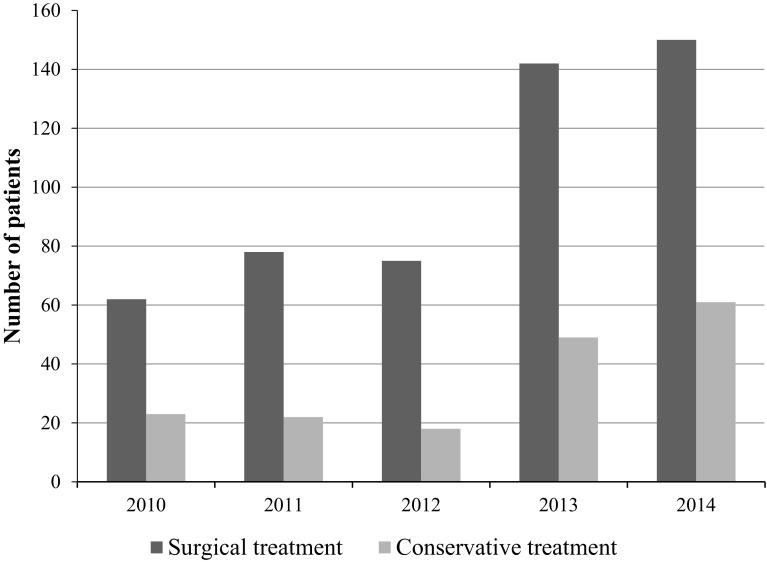
Development of treatments during study period

During the 5-year study period, the number of patients presenting with complications after penile self-injections increased; however, the percentage of conservative versus surgical treatments largely remained the same (Fig. 4, Table 4).

**Table 4:** Development of treatments during study period in numbers

Year	Patients	Percent
2010		
Surgical treatment	62	72.9
Conservative treatment	23	27.1
Total	85	100
2011		
Surgical treatment	78	78
Conservative treatment	22	22
Total	100	100
2012		
Surgical treatment	75	80.6
Conservative treatment	18	19.4
Total	93	100
2013		
Surgical treatment	142	74.3
Conservative treatment	49	25.7
Total	191	100
2014		
Surgical treatment	150	71.1
Conservative treatment	61	28.9
Total	211	100

The total patient load of The Trauma and Surgical Department at the Mae Tao Clinic has decreased during the latest years, while the number of cases of penile self-injections has increased (Fig. 4).

## Discussion

To our knowledge this study represents by far the largest series of complications to penile self-injections. We want to stress the importance of knowing symptoms related to penile self-injections, as patients presenting complications may be reluctant to inform about their previous self-injection. Taboo—illegal status of the procedure and expensive treatment not covered by health insurance—might be some of the reasons. Mae Tao Clinic offers free health-care to all patients and has established a renowned experience treating penile self-injections; this is probably the reason for the high number of cases seeking help at the clinic.

**Fig. 5:**
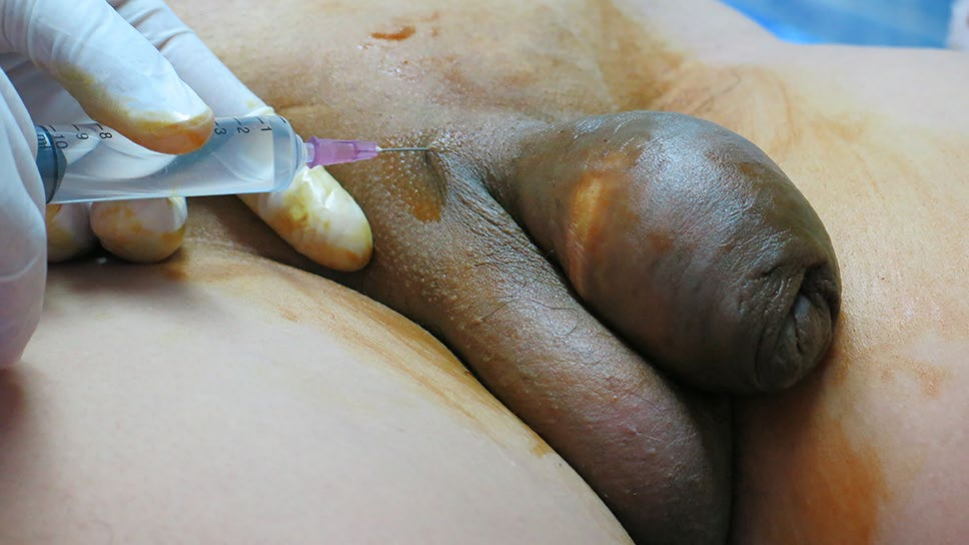
Pale penile skin colour change presented at the Mae Tao Clinic

At the time of presentation, nearly half of the included patients had severe complications in the form of indurated penile skin. We did not have the possibility to do histological examination of excised tissue, but macropathological examination done during surgery correlates with earlier studies documenting lipogranulomas as firm, disfigured, subcutaneous masses [5, 9, 14, 15]. In addition to these macropathological findings, skin colour change has been reported in a few cases in the available literature [14], and seen in 35 (5.1%) of our patients. Atrophy of the skin may be the underlying cause of the skin colour change (Fig. 5).

**Fig. 6:**
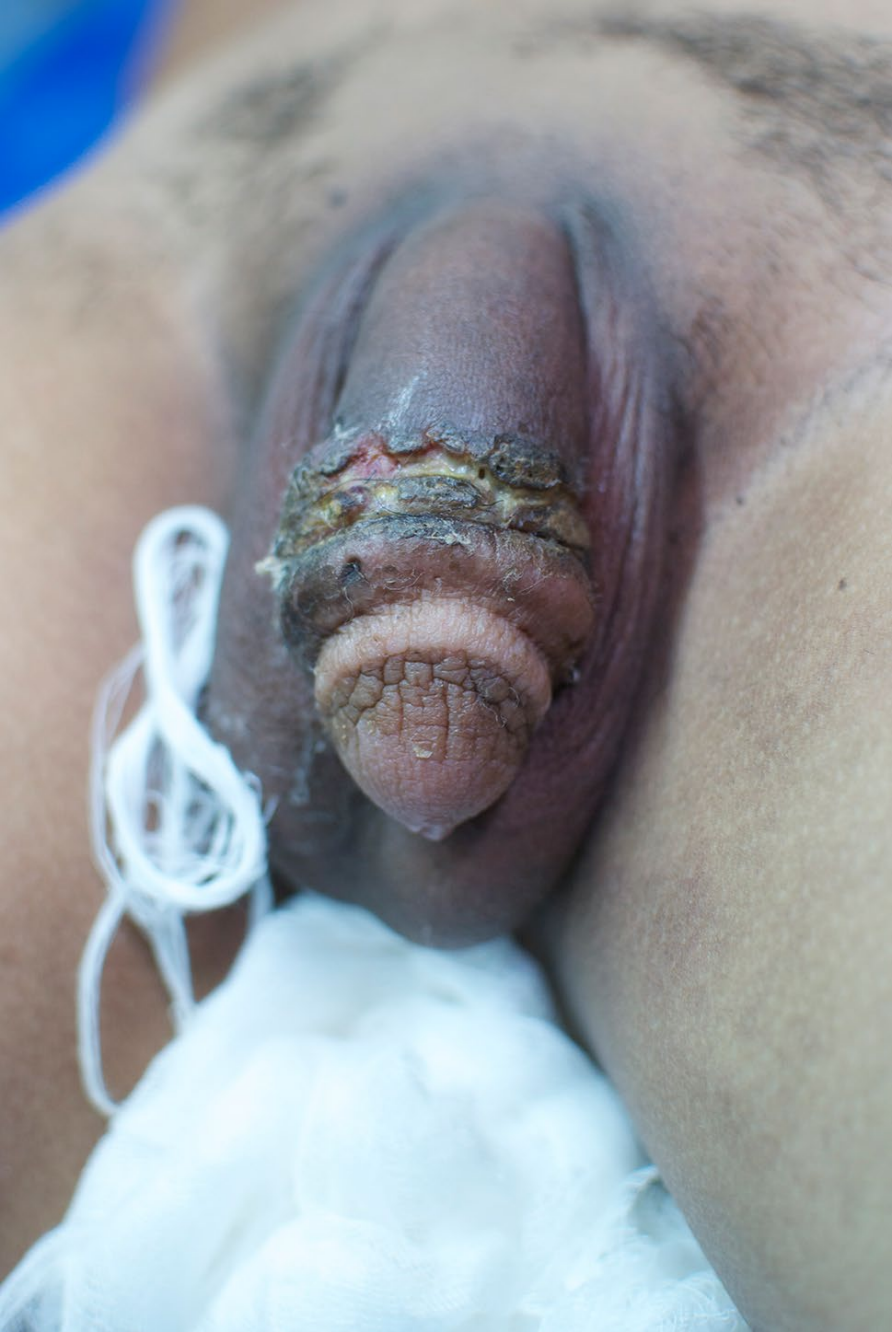
Ulcer presented at the Mae Tao Clinic

**Fig. 7:**
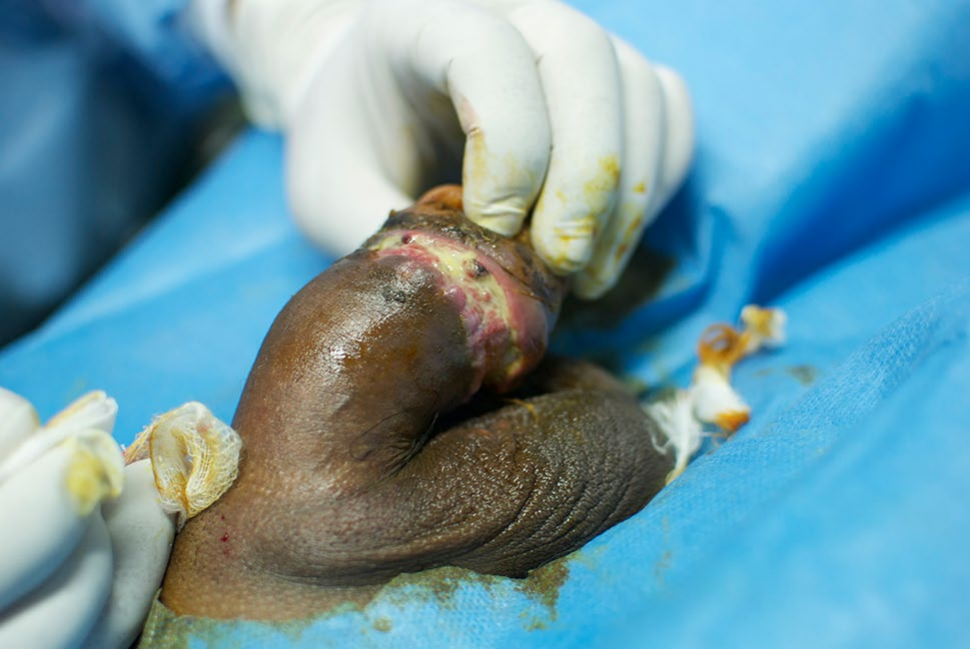
Ulcer presented at the Mae Tao Clinic

Some of our findings differ from earlier reports. Pain and swelling was seen in more than 80% of our patients. In an investigation of Korean prisoners (*N* = 357), only 15.5% reported pain [16]. We included painful erections in our pain category, which may explain the higher incidence of reported pain in our series. Furthermore, differences in pain score may be attributed to differences in the oil material injected. In another study of a prison population [12], in which 76 had performed penile self-injections with Vaseline (petrolatum), 17 (22.4%) reported pain, while as many as 40 (52.6%) had ulcerations. Compared to 12.8% in our findings (Figs. 6, 7). The high incidence of ulcers in the prison population may be explained by a setting of poor hygiene. In the earlier mentioned prison population described by Moon et al. [16] (*N* = 357), skin necrosis was reported in as many as 11.1%. It may be questioned if this is a direct result of penile injection or also related to lack of sterile equipment and poor hygiene.

Time of symptom presentation at the Mae Tao Clinic varied tremendously, ranging from a few days to 30 years after injection. This is in accordance with a case study by Eandi et al. [17], documenting a latency of complications of nearly 40 years. In two other studies, reporting on 26 [14] and 23 [18] cases, mean time from injection to presentation was 18.5 and 12.6 months, respectively. It has to be taken into consideration that the time between injection and hospitalization is not necessarily identical to the time between injection and initiation of complications. The majority of the men might be suspected to have been reluctant to seek medical help early in the course of the disease, due to the special taboo nature of this complex disease.

**Fig. 8:**
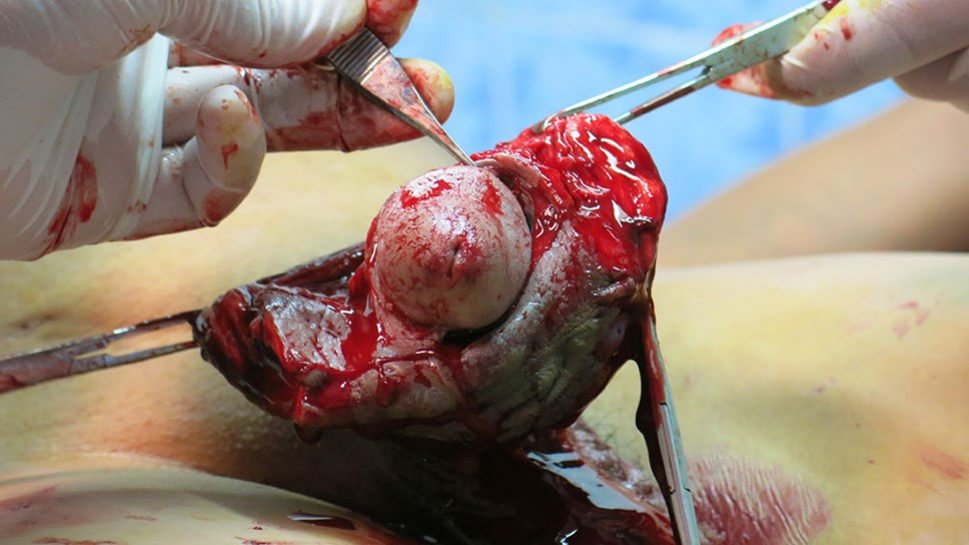
Full excision performed at the Mae Tao Clinic

**Fig. 9:**
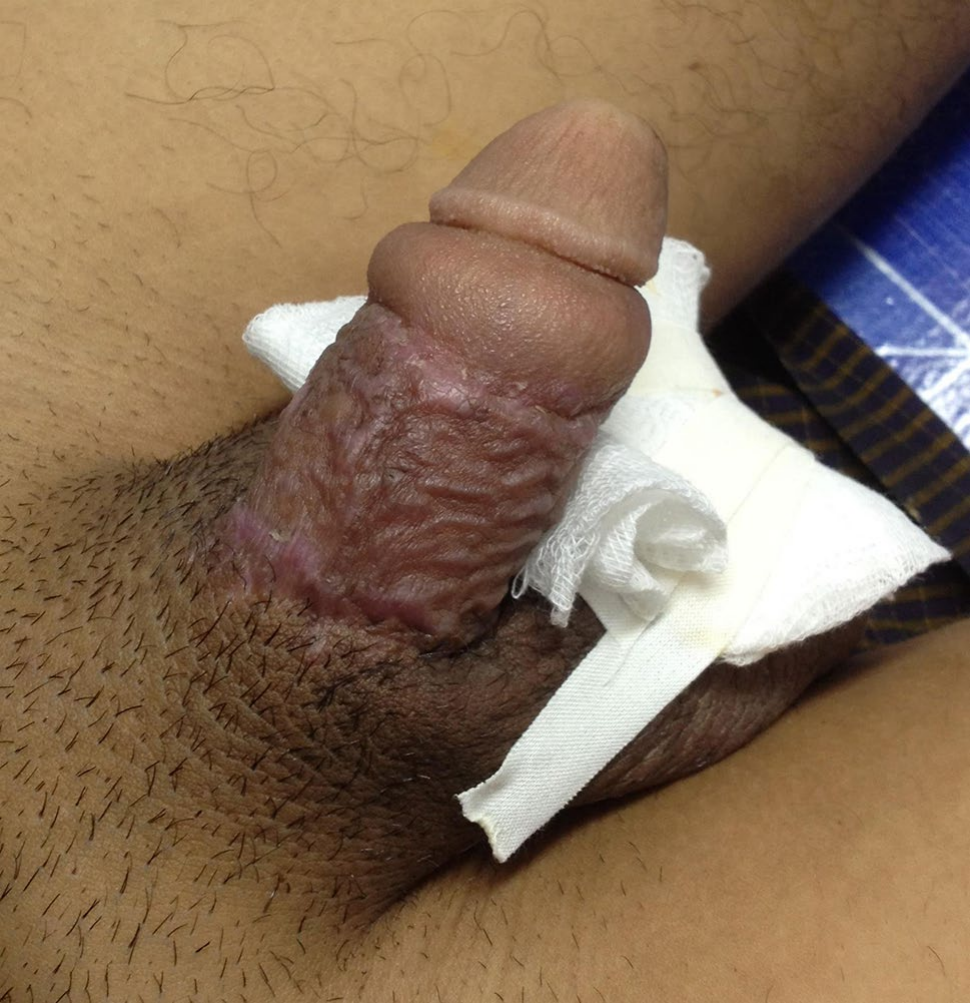
Excision and graft treatment performed at the Mae Tao Clinic

When the first case of complications following penile self-injection was seen at the Mae Tao Clinic in 2001, the clinic had no experience with surgical treatment of these complications. The surgical staff improvised with different kinds of conservative (antibiotics and/or dressings and/or analgesics) and surgical treatments (both partly removal and full excision of involved skin), but quickly learned that radical surgical excision of the involved skin followed by skin graft coverage was the only permanent reliable treatment (Fig. 8). This was confirmed in other published series [1, 2, 19]. Radical excision is recommended due to a high risk of subsequent serious complications, if not all foreign-body liquid is removed [6, 18, 20]. Skin coverage solutions involve split thickness skin graft, use of the prepuce (if not involved), scrotal skin flap [14, 21, 22], and Cecil’s inlay operation [10, 14]. In the two-staged Cecil’s operation the denuded penis is buried in scrotum and penoplasty is carried out 2–3 months later. Split thickness skin graft as used at the Mae Tao Clinic is a relatively simple technique, which has demonstrated good graft survival in the present series [6] (Fig. 9). Regardless of treatment option (conservative or surgical treatment) nearly all our patients were treated with antibiotics [637 of 680 patients (93.7%)]. It is the experience from the Mae Tao Clinic that antibiotics are mandatory for controlling secondary infections and as prophylaxis during surgical treatment. Since no follow-up data were available, we are not able to draw conclusions on treatment options. Even if failing as a permanent treatment, more research is needed on conservative options for patients that are reluctant to surgery for religious or subjective reasons, or when surgery is not a possible option [23]. There was no economical bias in choosing either conservative or surgical treatment options at the Mae Tao Clinic.

For prevention purposes, we registered the address of the patients to see if the frequency of injections was higher in certain areas. However, because some of the patients were immigrant workers and refugees, there was no consistency in patients giving home or temporary work addresses. Furthermore, many of the patients stayed undocumented in Thailand, which might have made them more reluctant giving their true address. What may be concluded from our observations is that, patients performing penile self-injections in this area obviously came from different locations and populations, and not just from one homogenous group as has been suggested in previous studies (e.g., fishermen [11], and prisoners [12, 16, 18]). Furthermore, concerning prevention, special consideration must be taken, as a wide spread informing-campaign in the general population potentially could lead to increased use of penile self-injections. Experience tells us that many of the patients got introduced to and underwent the procedure performed by non-medical personnel based on recommendations by acquaintances (data from interviews with surgical staff at the Mae Tao Clinic).

Risky sexual behaviour has been associated with penile oil self-injections in a study by Ohnmar et al. among Myanmar fishermen in Thailand (*N* = 48) [11]: 70.8% having had sex with commercial sex workers during the last year compared to 35.9% of men without injections (*N* = 440). Additionally, use of condoms was significantly lower in those with injections; only 8% always using condoms compared to 69% in the population without injections [11]. We did not find any signs suggesting that the patients studied at the Mae Tao Clinic were involved in risky sexual behaviour such as a higher prevalence of symptoms related to sexual transmitted diseases.

**Fig. 10:**
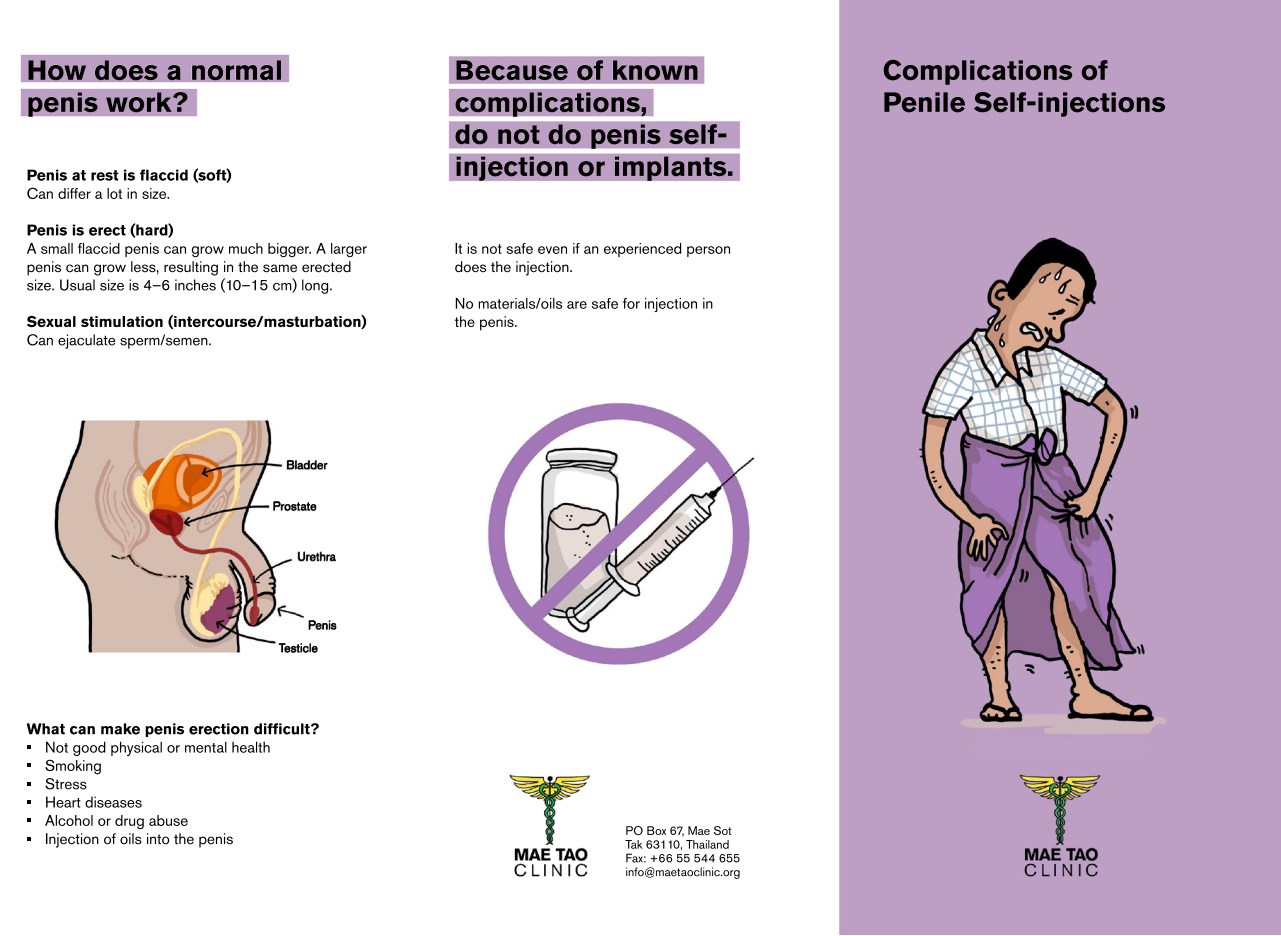

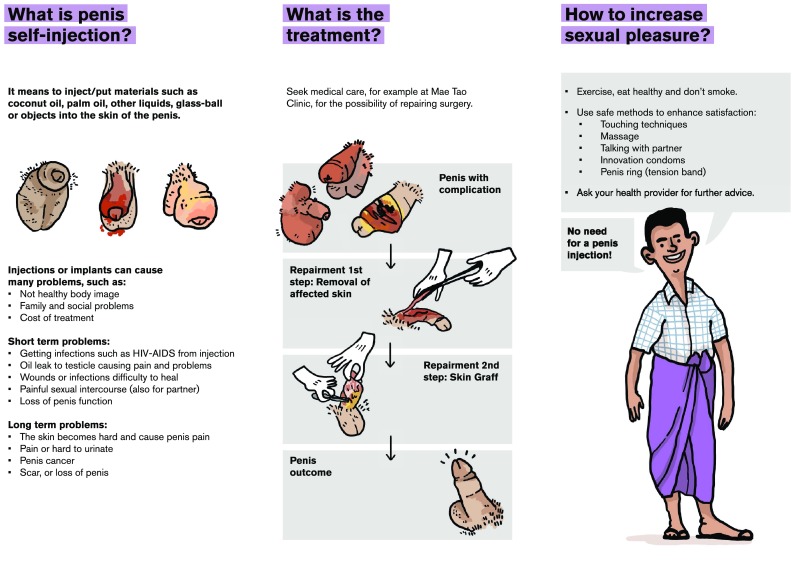
Information leaflet on complications of penile self-injections (English version)

Although this study has several limitations, including its retrospective design and the lack of systematic follow-up data, it is obvious that, penile oil self-injections do have serious health problems, and that the extent of the practice is probably more widespread in unlit areas than previously acknowledged. Our data should result in initiation of prevention campaigns directed both at risk populations and health-care professionals. Education of the latter about these practices and their debilitating and destructive consequences is essential, since patients often do not inform about the practice at their initial contact. Additionally, it is important to acknowledge and disseminate to risk populations that even severe complications may be treated successfully with radical excision of the lesions and skin grafts. Based on our data, prevention has been initiated in the Mae Tao Clinic (Fig. 10).

## Conclusion

Our data suggest that penile self-injections with mineral oil are more prevalent in certain areas than previously acknowledged. Our retrospective study showed that in 5 years more than 680 patients presented with complications to penile self-injections at the Mae Tao Clinic, of which 75% needed surgical intervention, mainly in the form of radical excision of the lesions followed by skin grafting. Preventive measures to this physically and psychologically devastating problem are highly warranted.
